# Molecular epidemiology of *Plasmodium falciparum* by multiplexed amplicon deep sequencing in Senegal

**DOI:** 10.1186/s12936-020-03471-7

**Published:** 2020-11-10

**Authors:** Tolla Ndiaye, Mouhamad Sy, Amy Gaye, Katherine J. Siddle, Daniel J. Park, Amy K. Bei, Awa B. Deme, Aminata Mbaye, Baba Dieye, Yaye Die Ndiaye, Ibrahima Mbaye Ndiaye, Mamadou Alpha Diallo, Khadim Diongue, Sarah K. Volkman, Aida Sadikh Badiane, Daouda Ndiaye

**Affiliations:** 1 Laboratoire de Parasitologie-Mycologie, Université Cheikh Anta Diop de Dakar (UCAD), Hôpital Aristide Le Dantec, Dakar, Senegal; 2 Broad Institute of MIT and Harvard, Cambridge, MA USA; 3 Yale School of Public Health, 60 College Street, New Haven, CT 06510 USA; 4 Department of Immunology and Infectious Diseases, Harvard University, Cambridge, MA USA

**Keywords:** Molecular epidemiology, *Plasmodium falciparum*, *Pfmsp1*, *Pfmsp2*, Multiplexed amplicon deep sequencing, MOI

## Abstract

**Background:**

Molecular epidemiology can provide important information regarding the genetic diversity and transmission of *Plasmodium falciparum*, which can assist in designing and monitoring elimination efforts. However, malaria molecular epidemiology including understanding the genetic diversity of the parasite and performing molecular surveillance of transmission has been poorly documented in Senegal. Next Generation Sequencing (NGS) offers a practical, fast and high-throughput approach to understand malaria population genetics. This study aims to unravel the population structure of *P. falciparum* and to estimate the allelic diversity, multiplicity of infection (MOI), and evolutionary patterns of the malaria parasite using the NGS platform.

**Methods:**

Multiplex amplicon deep sequencing of merozoite surface protein 1 (PfMSP1) and merozoite surface protein 2 (PfMSP2) in fifty-three *P. falciparum* isolates from two epidemiologically different areas in the South and North of Senegal, was carried out.

**Results:**

A total of 76 *Pfmsp1* and 116 *Pfmsp2* clones were identified and 135 different alleles were found, 56 and 79 belonged to the *pfmsp1* and *pfmsp2* genes, respectively. K1 and IC3D7 allelic families were most predominant in both sites. The local haplotype diversity (Hd) and nucleotide diversity (π) were higher in the South than in the North for both genes. For *pfmsp1*, a high positive Tajima’s D (TD) value was observed in the South (D = 2.0453) while negative TD value was recorded in the North (D = − 1.46045) and F-Statistic (Fst) was 0.19505. For *pfmsp2*, non-directional selection was found with a highly positive TD test in both areas and Fst was 0.02111. The mean MOI for both genes was 3.07 and 1.76 for the South and the North, respectively, with a statistically significant difference between areas (*p* = *0.001*).

**Conclusion:**

This study revealed a high genetic diversity of *pfmsp1* and *pfmsp2* genes and low genetic differentiation in *P. falciparum* population in Senegal. The MOI means were significantly different between the Southern and Northern areas. Findings also showed that multiplexed amplicon deep sequencing is a useful technique to investigate genetic diversity and molecular epidemiology of *P. falciparum* infections.

## Background

*Plasmodium falciparum* is the deadliest species of human malaria parasites (99.7%) and the most prevalent in Africa, responsible for 92% of malaria cases and 93% of death caused by malaria [1]. A better understanding of malaria epidemiology could be helpful for disease control and prevention. The extent of genetic diversity and multiplicity of infection (MOI) are essential to understand malaria epidemiological patterns, providing insight into the dynamics of malaria transmission, human exposure to mosquito bites, acquisition of malaria immunity [2–5] and assessing of malaria control interventions [6–8].

*Plasmodium falciparum* merozoite surface protein 1 (PfMSP1) and merozoite surface protein 2 (PfMSP2) are polymorphic antigens which have been extensively studied in malaria molecular epidemiology [9–11]. Polymerase chain reaction (PCR)-based genotyping of the polymorphic regions of the corresponding genes (block 2 of *Pfmsp1* and block 3 of *Pfmsp2*) can be used to assess the allelic diversity and to determine MOI [12]. Therefore, many previous studies have used these two markers in order to evaluate genetic diversity, population structure and MOI [13–16]. However, these PCR-based methods may underestimate the extent of allelic diversity in co-infected hosts due to the use of size-polymorphisms to infer alleles; a particular problem when using short fragment amplification [17–19]. Microsatellite typing is an alternative tool to assess genetic diversity but might fail to detect many minor clones as it requires a cut-off of 33% of the predominant peak for a minimal peak height [20–22]. Similarly, high resolution melting (HRM) has been used for genotyping of single nucleotide polymorphisms (SNP), but shows limitations in the detection of indels [23]. Advances in next generation sequencing (NGS) technologies and bioinformatic analysis has enabled the accurate detection of clones and even minor clones, overcoming the limitations of these other methods [24]. These techniques provide a more accurate estimation of MOI compared to the standard PCR-based methods [25, 26] and accurate assessment of genetic diversity in *P. falciparum* populations [27]. For example, amplicon deep sequencing of *Pfmsp1* from 222 samples collected in Ethiopia revealed 307 haplotypes out of which 99 were predominant haplotypes (the clone had the highest frequency within infection). In addition, a mean MOI of 2.68 was found by this study [24].

In Senegal, most of the studies about *P. falciparum* genetic diversity and malaria molecular epidemiology [7, 8, 28–30] have been carried out in the Thies region, a low malaria endemicity area. Thus, limited information on malaria molecular epidemiology are available for other areas of the country and further detailed studies are needed to understand the genetic diversity and population structure country-wide. Here, multiplexed amplicon deep sequencing *of Pfmsp1* and *Pfmsp2* genes in *P. falciparum* isolates sampled from a malaria hyper-endemic area in the South (Kedougou region) and a pre-elimination areas in the North (Podor and Matam) was performed. This data was used to understand the population structure, to estimate allelic diversities and MOI, and to understand the population dynamics, gene flow and evolutionary pattern of the malaria parasite *P. falciparum* in Senegal.

## Methods

### Study sites, sample collection

**Fig. 1:**
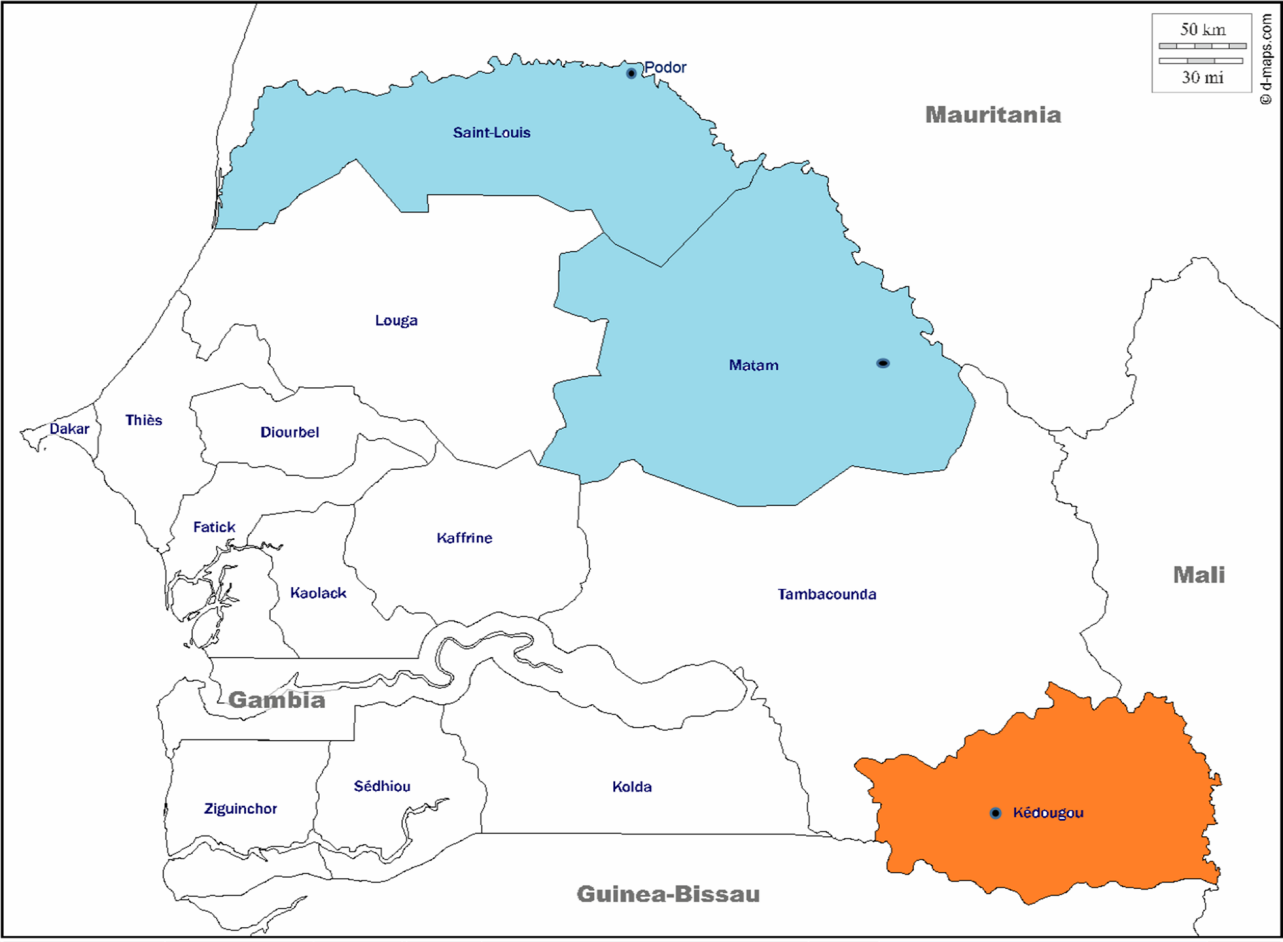
Map of Senegal showing the two malaria studies areas, Kedougou in orange (Southern Senegal) and Podor and Matam in blue (Northern Senegal). This map was generated using online website (https://www.d-maps.com)

This study was conducted in Kédougou region, (KG, Southern Senegal) and Podor and Matam (PM, Northern Senegal). Kédougou (685 km from the capital, Dakar) (Fig. 1) is hyper-endemic for malaria with an incidence higher than fifteen malaria cases per 1000 habitants. In this area, malaria transmission is seasonal from July to December with a high entomological inoculation rate (EIR) ranging from 20 to 100 infectious bites/person/ year [31]. In contrast, Northern Senegal (Podor and Matam, Fig. 1) is a malaria pre-elimination area with an incidence of less than five cases per 1,000 habitants and an EIR equal to 1.3 infectious bites/person/ year [31].

Samples were collected from patients with malaria symptoms attending the National Malaria Control Programme (NMCP) sentinel site health facilities during malaria transmission season between September and December 2016 (Fig. 1). In the South, venous blood samples were collected on filter paper from patients fulfilling the following inclusion criteria: having fever (axillary temperature ≥ 37.5 °C) or history of fever in the previous 48 h, age ranging from 6 months to 75 years and suffering from uncomplicated *P*. *falciparum* malaria with parasite density ≥ 1000 asexual forms per microlitre. Patients who presented signs or symptoms of severe malaria as defined by the World Health Organization (WHO) [32] and pregnant women were not included in the study. In the North, *P. falciparum* positive rapid diagnostic tests (RDTs) were collected in health facilities (NMCP sentinel sites) in Matam and Podor.

### Amplification and sequence analysis of *Pfmsp1* and *Pfmsp2*

**Fig. 2:**
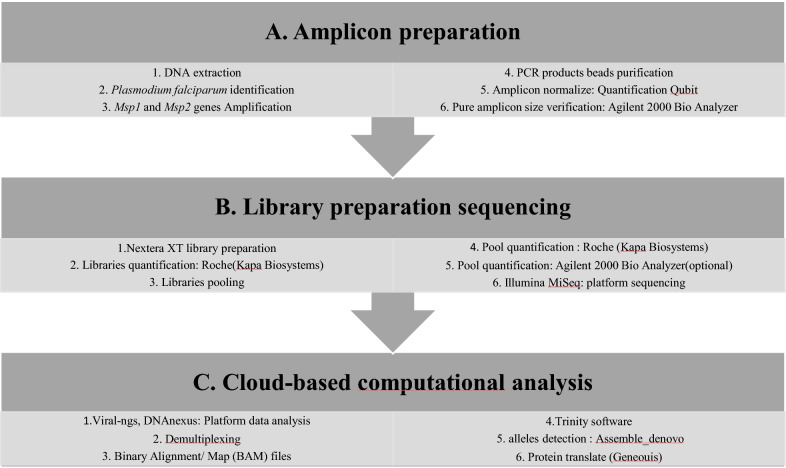
Experimental workflow from amplicon preparation to data analysis with three steps: amplicon preparation (**a**), library preparation and sequencing (**b**) and sequences data analysis (**c**)

The experimental workflow from amplicon preparation to data analysis are detailed in Fig. 2.

### Multiplex PCR amplification of *Pfmsp1* and *Pfmsp2* genes

Parasite genomic deoxyribonucleic acid (DNA), from filter paper and RDTs was extracted using QIAamp DNA Mini kit (Qiagen, QIAGEN, USA) according to the manufacturer's instructions. *Plasmodium falciparum* molecular identification was performed using the photo-induced electron transfer (PET)-PCR assay [33] on a Roche LightCycler 96 instrument (Roche Molecular Systems, Inc). Each experimental run included both a negative (no template) and a positive (3D7 *P. falciparum* strain) control. Samples with a cycle threshold (CT) of 40 or less were scored as positive [33, 34]. The two *Pfmsp1* and *Pfmsp2* polymorphic genes were amplified by multiplex PCR. All PCR reactions were conducted in a 20 μl reaction mixture containing 2 μl of template DNA, 4 μl Phusion high-fidelity (HF) PCR master mix, 0.2 μl HF Phusion Taq Polymerase, 250 μM deoxyribonucleotide triphosphate (dNTPs), 0.5 μM of each forward and reverse primer of each gene. Cycling conditions were as follows: initial denaturation at 94 °C for 5 min, followed by 40 cycles of denaturation at 94 °C for 30 s, annealing at 58 °C for 1 min and extension at 68 °C for 1 min 40 s; a final extension was done at 68 °C for 5 min [23, 35]. The PCR products were revealed by electrophoresis on 2% agarose gels stained with ethidium bromide and visualized under ultraviolet (UV) trans-illumination (VersaDoc®, BIORAD, Hercules, USA). The size of PCR fragments was estimated using a 100 bp molecular weight ladder (Fig. 2a). The length of PCR product varied from 800–1000 bp and from 600 to 800 bp for *Pfmsp1* and *Pfmsp2*, respectively.

### Multiplex amplicon deep sequencing of *Pfmsp1 *and *Pfmsp2*

Amplicons from each sample were purified using a 0.6X DNA SPRI (Agencourt AMPure XP beads Beckman Coulter ®, CA, USA). Samples were normalized to 0.25 ng/μl concentration using the Qubit® 3.0 Fluorometer and Invitrogen™ Qubit® Quantitation Kit (Life Technologies, Carlsbad, California). The Nextera XT DNA Library Preparation kit (Illumina, USA) was used for library preparation of 1 ng of purified PCR product from each sample [36]. Sequencing libraries were cleaned using a 0.6X DNA SPRI and quantified by qPCR using the KAPA Library Quantification kit (KapaBiosystems, MA, USA) on a Roche LightCycler 96 instrument. The concentration of sequencing libraries was normalized and all samples were pooled. The concentration and mean fragment length of the pool were determined using the Agilent High Sensitivity DNA Kit (2100 Expert Software) and the concentration was confirmed by KAPA qPCR. The pool of all samples was sequenced on one run of an Illumina Miseq sequencer with a Miseq v2 reagent Kit (VEROGEN, North America) and 100nt paired end sequencing (Fig. 2b).

### Data analysis

Sequencing data was analysed using viral-ngs, version 1.19.2 pipeline implemented on the DNAnexus cloud-based platform [37]. Briefly, raw reads were demultiplexed to individual sample libraries and reads mapping to the human genome or to other known technical contaminants (e.g., sequencing adapters) were removed. After filtering the reads, they were assembled based on *Pfmsp1* or *Pfmsp2* gene mapping using the Trinity software [38]. For every sample, the number of clones of each gene were determined from the number of contigs generated by Trinity software. Following de-novo assembly, clones were filtered considering an average of k-mer coverage of 2 with length of at least 200 bp [2*(k − 1), k = 101]. The average read coverage of the individual contig was 1000×, the percentage of the average read coverage of individual contig was 1% of the average read coverage of overall contigs and allele frequencies > 1%. The clones found were classified into allelic families according to the polymorphism of block 2 of *Pfmsp1*, and block 3 of *Pfmsp2* using the Geneious software (Fig. 2c). For *Pfmsp1*, K1-like were characterized by amino acid tripeptide repeat units SAQ, SGT, SGA, SGP, MAD20-like by SVA, SGG, SKG, SVT and RO33-like by non-synonymous amino acid change. For *Pfmsp2*, IC3D7-like presented GA or SG amino acid dipeptides repeat units and a poly-threonine repeat while FC27-like were characterized by 32 amino acid, ADTIASGSQSSTNSASTSTTNNGESQTTTPTA or its variants and 12 amino acid: ESISPSPPITTT.

For genetic diversity and population structure analysis, DNA sequences were first aligned using BioEdit Sequence Alignment Editor V.7.0.5.3 [39]. DnaSP v. 6.0 software was used to estimate haplotype diversity (Hd), nucleotide diversity (π) and Tajima's D (TD) [40, 41]. Genetic differentiation between populations, Wright’s F-statistic (Fst), was estimated using Arlequin software version 3.5 [42]. The Geneious software was used to create the neighbour-joining tree in order to identify the number of parasite clusters and parental links between strains.

The MOI mean was calculated by dividing the total number of alleles detected by the total number of samples for each gene [43]. The online Biostatgv Student’s test (https://biostatgv.sentiweb.fr/?module=tests/student) was used to compare MOI between localities. For all tests, comparisons were considered statistically significant when P-values < 0.05.

## Results

Fifty-three (53) samples (28 from the South and 25 from the North) were successfully amplified and sequenced for the two polymorphic markers *Pfmsp1* and *Pfmsp2.* All Fastq sequencing data were submitted to the SRA/NCBI database with the following accession numbers: from SRX8632497 to SRX8632549 (https://www.ncbi.nlm.nih.gov/sra/PRJNA642844).

### Allelic polymorphisms and population structure of *Pfmsp1*

**Fig. 3:**
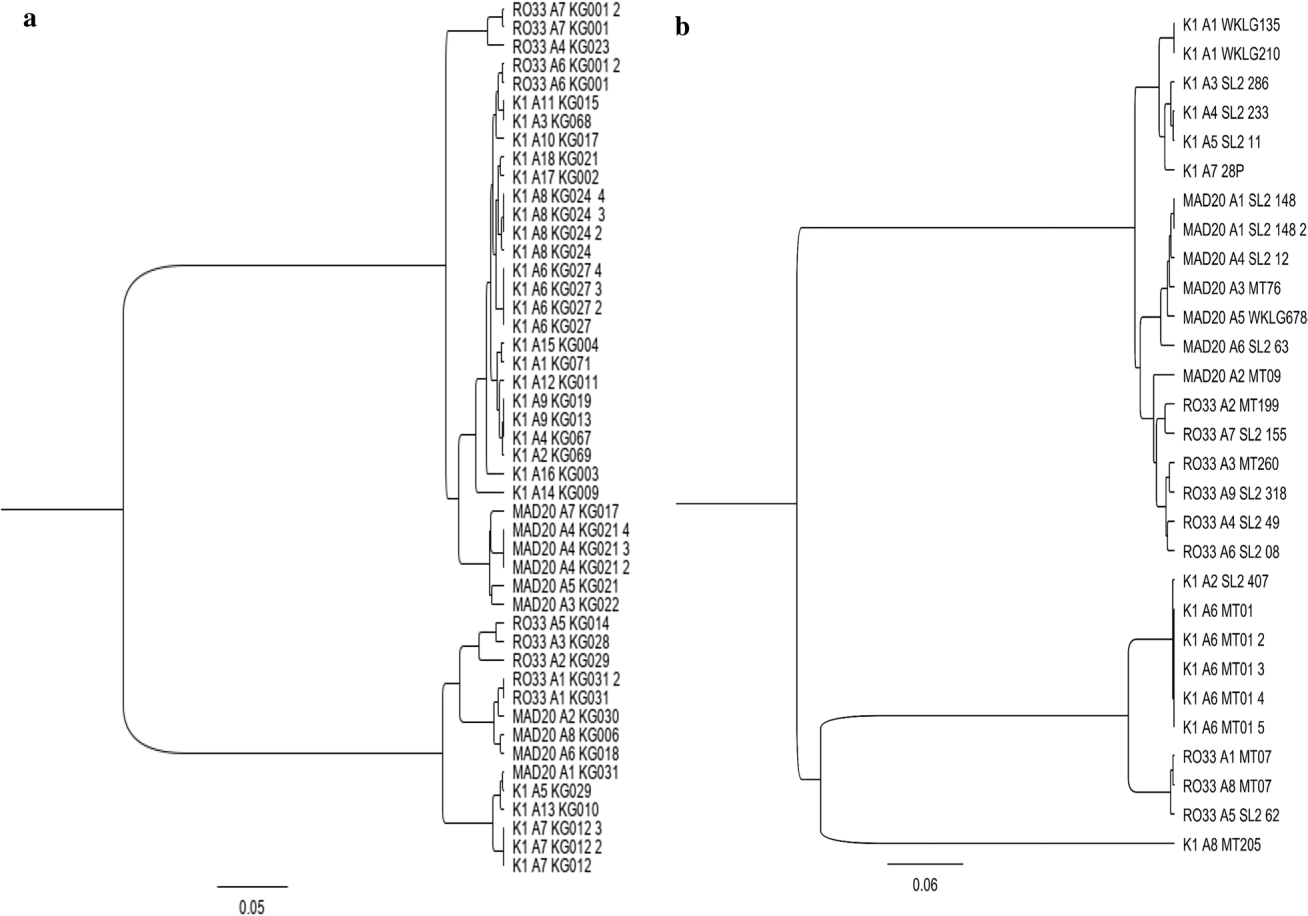
Neighbor-joining tree of K1, MAD20 and RO33 allele types of *msp1* gene of 53 *P.falciparum* isolates. **a** Neighbor-joining tree showing the genetic relatedness between *msp1* alleles in samples from the South. **b** Neighbor-joining tree showing the genetic relatedness between *msp1* alleles in samples from the North

Fifty-one *Pfmsp1* gene were successfully assembled from 53 *P. falciparum* isolates. A total of 76 clones were found of which 47 and 29 in the South and the North, respectively (Additional file 1). Of the detected *Pfmsp1* clones, 56 different alleles were identified with 33 in the South area and 23 in the North area. In the South, the 33 alleles found were classified into 3 different allele types: 18 of K1-like, 8 of MAD20-like, and 7 of RO33-like (Table 1). In the K1-like, each allele had different numbers and arrangements of amino acid tripeptide repeat unit SGASAQSGT, SGT or SGPSGT. The MAD20-like were varied by the number of SGGSVA amino acid tripeptide motif. Meanwhile RO33-like was less polymorphic, with only limited numbers of amino acid substitutions. The neighbor-joining tree of different allele types showed two parasite clusters (Fig. 3a). *Plasmodium falciparum* population presented high level of genetic diversity with high haplotype diversity (Hd) of 0.93 and a nucleotide diversity (π) of 0.28306. The TD test was performed in each area in order to identify any deviation from the neutral evolution of parasites and a highly positive TD value was observed in the South (D = 2.0453) with a statistically significantly different (p < 0.05) (Table 2).

**Table 1:** number of alleles per allelic family of *Pfmsp1* and *Pfmsp2* in the South (Kedougou) and the North (Podor and Matam)

Localities	Kedougou	North area
Gene/allelic families	N	
Msp1		
K1-like	18	8
MAD20-like	8	6
RO33-like	7	9
Total alleles	33	23
Msp2		
IC/3D7-like	35	19
FC27-like	18	7
Total alleles	53	26

*N* number of alleles

**Table 2:** Genetic diversity of *msp1* and *msp2* genes of *P. falciparum* isolates from the South (Kedougou) and North (Podor and Matam)

Genes	*Msp1*		*Msp2*	
	Kedougou	North area	Kedougou	North area
No of Isolate	28	23	28	25
Clones (N)	47	29	76	40
Haplotype diversity (Hd)	0.93	0.761	0.82	0.843
Nucleotide diversity				
π	0.28306	0.09457	0.48339	0.42473
θ	0.24245	0.16236	0.3534	0.37802
Tajima's D⁎ test of neutrality	2.0453*	− 1.46045	5.21073*	3.46684*
FSTs	0.19505*		0.02111	

^*^indicates the significance at P 0.05 level

In the North, the 23 alleles found were classified into: 8 of K1-like, 6 of MAD20-like and 9 of RO33-like (Table 1). The K1-like were different based on the number of amino acid tripeptide repetition SGASAQSGT or SGPSGT. The MAD20-like varied with SGGSVA repeat unit and RO33-like had relatively well conserved sequences. The neighbour-joining tree of different allele types revealed two parasites clusters (Fig. 3b). Parasite population exhibited less genetic variation with π = 0.09457 and Hd = 0.76 and a negative TD value was recorded (D = -1.46045), which was not statistically significant (Table 2). A significant genetic differentiation between the South and the North *P. falciparum* populations was observed with Fst = 0.19505 (*p* < *0.05*) (Table 2).

### Allelic polymorphisms and population structure of *Pfmsp2*

All 53 *Pfmsp2* gene of *P. falciparum* isolates were successfully assembled. A total of 116 clones were found, 76 in the South and 40 in the North (Additional file 2). Among these clones 79 different alleles were identified of which 53 and 26 in the South and the North, respectively.

**Fig. 4:**
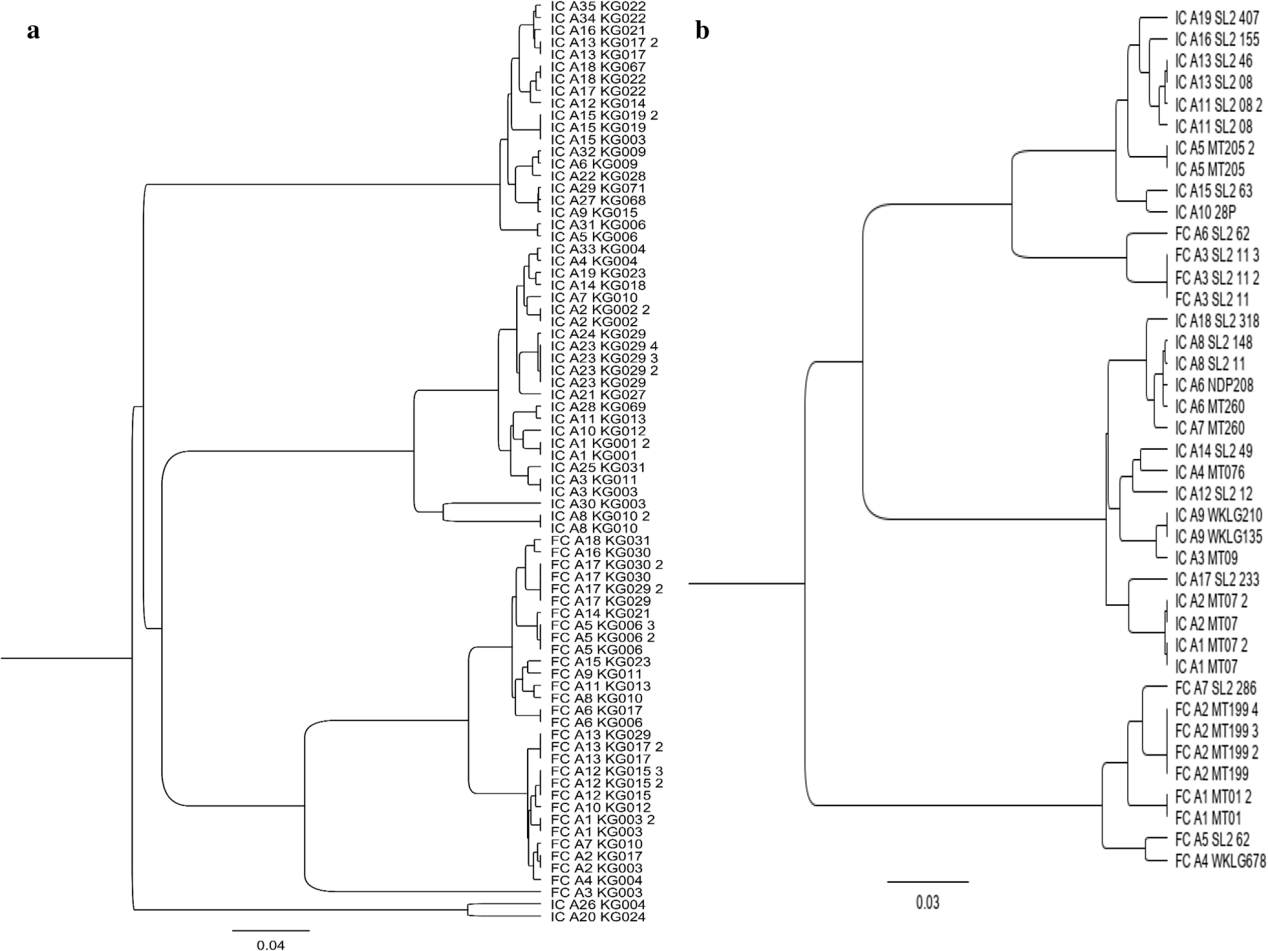
Neighbor-joining tree of IC3D7 and FC27 allele types of *msp2* gene of 53 *P.falciparum* isolates. **a** Neighbor-joining tree showing the genetic relatedness between *msp2* alleles in samples from the South. **b** Neighbor-joining tree showing the genetic relatedness between *msp2* alleles in samples from the North

In the South, among the 53 different alleles detected, 35 belonged to the IC3D7-like while the other 18 were classified into the FC27-like (Table 1). IC3D7-like showed different dimorphic structures based on GA or SG amino acid dipeptides repeat units and poly-threonine repeats. Meanwhile, FC27-like varied with (ADTIASGSQSSTNSASTSTTNNGESQTTTPTA) or its variant amino acid succession. The neighbour-joining tree of parasites showed four clusters (Fig. 4a).

In the North, of the detected 26 different alleles, 19 were classified into IC3D7-like and 7 belonged to FC27-like (Table 1). IC3D7-like varied with GA or SG amino acid dipeptides repeat units and poly-threonine repeat. FC27-like were different based on the amino acid succession (ADTIASGSQSSTNSASTSTTNNGESQTTTPTA) and the typical non-synonymous substitution of 6 amino acids (SSGNAP) was found at the C-terminal region of 5 of the 7 FC27-like identified. The neighbour-joining tree of different alleles revealed three clusters (Fig. 4b).

The observed genetic diversity was similarly elevated in both regions (Hd = 0.82 in the South and Hd = 0.843 in the North), and nucleotide diversity was also very similar (π = 0.48339 in the South and π = 0.42473 in the North). Non-directional selection was found with a highly positive TD test in the two areas, D = 5.21073 in the South and D = 3.46684 in the North with statistically different (*p* < *0.05*). The Fst between the two localities showed an almost panmictic population (Fst = 0.02111) (Table 2).

### Multiplicity of infection (MOI)

**Fig. 5:**
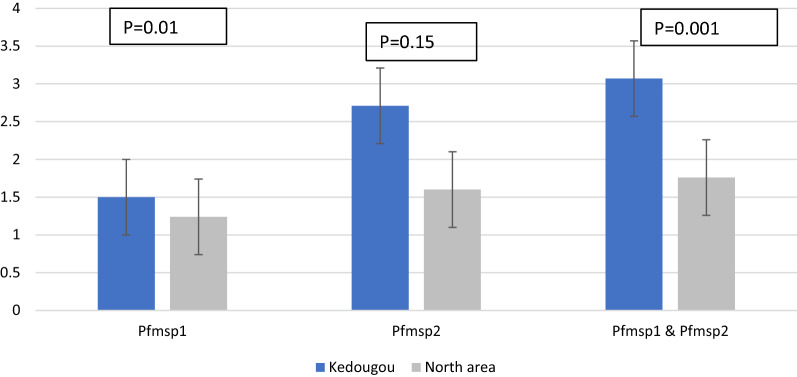
*Plasmodium falciparum* multiplicity of infections (MOI) mean for *Pfmsp1* and *Pfmsp2* genes in the South area and North area. The MOI mean of *Pfmsp1* & *Pfmsp2* was statistically different between the South and North (*p* = 0.001)

The mean MOI for *Pfmsp1*, *Pfmsp2* and both genes together in each area are shown in Fig. 5. The mean MOI for *Pfmsp1* was 1.5 in the South and 1.24 in the North, the number of clones per isolate ranged from 1 to 5 in these two regions and no statistically significant difference was observed between regions (*p* = *0.159*). For *Pfmsp2*, mean MOI was significantly different between the South and the North (*p* = *0.010*). The number of clones per samples ranged from 1 to 7 in the South with a MOI = 2.71 while in the North a MOI = 1.6 and the average number of clones varying between 1 and 4. Mean MOI for both genes was 3.07 and 1.76 in the South and North, respectively, with statistically significant differences between areas (*p* = *0.001).*

## Discussion

A better understanding of *P. falciparum* population structure could help to improve the local monitoring of parasite transmission, particularly in areas where *P. falciparum* genetic diversity has been poorly documented. Therefore, the aim of this study was to report the genetic diversity of *P. falciparum* and parasite population structure by performing multiplexed amplicon deep sequencing of *Pfmsp1* and *Pfmsp2* from two understudied areas in Senegal with significantly different endemicity, the Southern (malaria hyper-endemic) and Northern (malaria pre-elimination) areas. A high degree of polymorphism of the genes *Pfmsp1* and *Pfmsp2* was found in Senegal. A total of one hundred thirty-five different alleles were identified; 56 *Pfmsp1* alleles (26 K1-like, 14 MAD20-like and 16 RO33-like) and 79 *Pfmsp2* alleles (54 IC3D7-like and 25 FC27-like). These results are consistent with a previous observation of high levels of polymorphism in both *Pfmsp1* (8K1-like, 14MAD20-like, 6 RO33-like) and *Pfmsp2* (41 IC3D7-like, 18 FC27-like) using an amplicon deep sequencing technique in Myanmar [44]. Similar results have been also reported by Aspeling-Jones et al. who identified 225 different K1-like, 123 different MAD20-like and 9 distinct RO33-like in Africa and Asia [45]. Most of these alleles were detected in the South, hyper-endemic area, 33 and 53 distinct alleles were observed for *Pfmsp1* (18 K1-like, 8 MAD20-like, and 7 RO33-like) and *Pfmsp2* (35 IC3D7-like and 18 FC27-like), respectively. Comparable results from elsewhere in sub-Saharan Africa (Gabon and Ivory-Cost) reported by Yavo et al*.* underlined the same diversity with 27 K1-like, 22 MAD20-like and 18 RO33-like alleles for *Pfmsp1* and 28 IC3D7-like and 20 FC27-like for *Pfmsp2* [16]. In areas with high malaria transmission intensity the probability of genetic recombination between circulating strains is high and could affected the variation, the numbers and arrangements of amino acid repeats units of *Pfmsp1* and *Pfmsp2,* which are the main factors driving the increased allelic diversity [44, 46–48]. It is known that a large number of mutations in parasite populations arms the organism to successfully battle out adverse environmental conditions through adaptation. Indeed, it has been shown that *P. falciparum* genetic diversity is indicative of the ability of malaria parasites to adapt to their hosts by selection of advantageous traits, such as drug resistance and antigenic variability [49].

For *Pfmsp1*, despite a haplotype diversity of Hd = 0.76 in the North, the negative value of TD suggests there is clonal expansion of the parasite. A similar finding was also reported by the team of Daniels et al*.* in 2013 in Thiès, using a 24 SNP molecular barcode approach [30]. This situation suggests a high self-fertilization rate between genetically identical parasites during the sexual stages in the mosquito in these areas where malaria transmission is declining. However, in the South the *P. falciparum* population exhibits a high Hd (Hd = 0.93) and a TD value significantly different from expected under the neutral model of molecular evolution (D = 2.0453). This variability in parasite population genetics observed across the two regions reflected the difference in malaria transmission intensity reported in these areas [2]. These findings suggest that the genetic diversity of *P. falciparum* is greater in high malaria transmission areas and decreases when transmission regresses [2, 4]. The neighbour-joining tree of different allele types revealed two parasite clusters in the South as in the North and many clones sharing same patterns in amino acid level showing parental link between strains. Finally, the low Fst value of *Pfmsp1* between parasite population from the South and the North indicates the presence of gene flow between these populations, likely facilitated by extensive human migration events between regions causing the vector’s displacement.

For *Pfmsp2*, three clusters with a high Hd and π were observed associated with a balancing selection (Positive TD value) in each area and an almost panmictic parasite population (low Fst and genetic relatedness) between the two studies sites. As widely known, malaria parasites use genetic diversity as an escape measure either from human immunity or treatment by anti-malarials [50, 51]. Therefore the high genetic diversity of parasites found in these areas is alarming. Indeed, a larger number of different allele types in the parasite population may be able help parasites more easily adapt to the environmental conditions such as malaria control measures [52, 53]. In addition, the low genetic differentiation between the parasite populations from the two study sites indicates a gene flow within the population probably deriving from the high mobility of infected human hosts. This situation has been observed to cause malaria outbreaks in malaria residual foci (unpublished data) and a rebound of *P. falciparum* genetic diversity in the low malaria transmission region of Thies, as previously reported [7]. Such events could be a major challenge for malaria control and elimination in Senegal. Multiclonal infections were higher in the South, suggesting that this is more common in areas of high malaria endemicity. This is consistent with previous observations for high transmission areas, such as in Ethiopia [24]. Allelic polymorphism was also higher in the South, which could increase the likelihood of recombination (genetic crossing-over) in these areas [46]. Recombination can generate novel parasite variants, giving rise to new genetic diversity that may exhibit phenotypic differences such as virulence, drug resistance or immune evasion [54, 55]. MOI is an indicator of malaria transmission level because: it has been found to be higher in high malaria transmission areas and decreases when transmission declines [2, 56–61]. MOI mean was significantly higher in the South than in the North (*p* = *0.001*), suggesting that malaria transmission remains still active in this area. Moreover, the low mean MOI found in the North (1.76), may suggest a decline in malaria transmission levels, underlining the effectiveness of the scale-up in malaria control measures since 2006 [32].

This study was carried out from malaria symptomatic patients. As known, in endemic areas asymptomatic malaria or sub-microscopic infections is thought to represent the majority of the infections [62]. Therefore, missing data about parasites genetic diversity from these asymptomatic patients may constitute a limitation of this study. Despite this limitation, the results of this study contribute insight into the genetic diversity of the *P. falciparum* population in two malaria endemic areas in Senegal. In addition, this study argues that multiplexed amplicon deep sequencing represents an important advance for surveillance of parasites populations within the country. Although this approach is currently more expensive and longer per-sample that standard genotyping methods it offers several advantages, especially in the ability to scale to large numbers of samples as will be needed for large-scale disease surveillance. The costs of sequencing are also rapidly declining and methods are becoming faster and more portable. Finally, this method allows the sequencing of several genes of interest in one run and can be readily adapted to other genes as the sequencing of anti-malaria resistance markers.

## Conclusion

This study revealed a high genetic diversity of *Pfmsp1* and *Pfmsp2* genes in Senegal. Findings also showed an almost panmictic *P. falciparum* population with an important gene flow and MOI disparities between a malaria hotspot (Kedougou, Southern area) and a malaria pre-elimination area (Podor and Matam, Northern areas), a situation that may hamper malaria elimination in these Northern regions. Therefore, continuous molecular epidemiological surveillance to monitor the genetic diversity and parasite population structure in Senegal would be necessary for malaria control in Senegal. Additionally, this study showed that multiplexed amplicon deep sequencing is a useful technique to investigate genetic diversity and molecular epidemiology of *P. falciparum* parasite infections.

## Supplementary information

**Additional file 1.** Multiplexed amplicon deep sequencing data of Pfmsp1 gene.

**Additional file 2.** Multiplexed amplicon deep sequencing data of Pfmsp2 gene.

## Data Availability

The data supporting the findings of this article are included within the article.

## Abbreviations

NGS: Next generation sequencing
MOI: Multiplicity of infection
PfMSP1: Merozoite surface protein 1 of *Plasmodium falciparum*
PfMSP2: Merozoite surface protein 2 of *Plasmodium falciparum*
*pfmsp1*: *Plasmodium falciparum msp1* Gene
*pfmsp2*: *Plasmodium falciparum msp2* Gene
Hd: Haplotype diversity
π: Nucleotide diversity
TD: Tajima’s D
PCR: Polymerase chain reaction
HRM: High resolution melting
SNP: Single nucleotide polymorphism
NMCP: National Malaria Control Programme
WHO: World Health Organization
EIR: Entomological inoculation rate
DNA: Deoxyribonucleic acid
RDTs: Rapid Diagnostic Tests
PET: Photo-induced electron transfer
CT: Cycle threshold
HF: High-fidelity
DNTPs: Deoxyribonucleotide triphosphate
UV: Ultraviolet
Fst: F-Statistic
NCBI: National Center for Biotechnology Information
PM: Podor and Matam
KG: Kédougou
